# Oxidative Stress Indices as Markers of Lead and Cadmium Exposure Toxicity in Auto Technicians in Ibadan, Nigeria

**DOI:** 10.1155/2019/3030614

**Published:** 2019-08-21

**Authors:** Ishiaq Olayinka Omotosho

**Affiliations:** Department of Chemical Pathology, University of Ibadan, Nigeria

## Abstract

Auto technicians (auto mechanics, panel beaters, battery chargers, and auto painters) are among the most valuable work force in the society. Reports on oxidative stress in persons occupationally exposed to mixed chemicals abound; however, few have narrowed down specifically on auto technicians, while even fewer have stratified the exposure in the different subgroups of auto technicians. This study evaluated the antioxidant status in auto technicians routinely exposed to lead and cadmium and stratified the results of exposure by different subgroups of auto technicians in Ibadan, Nigeria. Sixty-five apparently healthy males (aged 18 to 65years) were selected based on specific inclusion criteria using a structured questionnaire. Thirty-four were cases consisting of participants routinely working as auto technicians or apprentices(≥2years) while controls were thirty-one nonoccupationally exposed male members of staff/students of the University College Hospital, Ibadan, Nigeria. Blood was collected from all participants and analyzed for the presence of lead, total antioxidant capacity (TAC), and total plasma peroxides (TPP); oxidative stress index (OSI) was calculated. Urine samples collected from all participants were analyzed for the presence of urinary lead and cadmium using standard laboratory methods. Although values of TAC in cases (22538 ± 8726.54) were not statistically different from what was obtained in controls (26741.87 ± 8696.68), TPP and OSI were statistically higher in cases than in controls (183.88 ± 53.39 and 120.16 ± 70.54, respectively, and 0.93 ± 0.45 and 0.49 ± 0.33, respectively). The blood lead level in cases (10.11 ± 4.47) was significantly higher than in controls (7.72 ± 1.22) while elevated urinary lead and cadmium levels were observed in cases (0.65 ± 0.21 and 0.34 ± 0.11, respectively) compared to controls (0.52 ± 0.19 and 0.27 ± 0.10, respectively). Raised TPP and OSI levels—hallmark of active lipid peroxidation—found to be highest among panel beaters compared to others may be prognostic of membrane-damaging diseases in this subgroup of auto technicians.

## 1. Introduction

Automobile technicians are among the group of artisans most vulnerable to occupational and environmental pollution. They include auto mechanics, automobile body repairers (panel beaters), auto painters, welders, vulcanizers, and auto electricians who are all working on different areas of an automobile. Notable among many environmental pollutants in the automobile workplace is exposure to lead and cadmium. The greatest problem of exposure to these pollutants in this kind of setting is their low but continuous exposure which on a long term produces chronic toxicity. This is worsened particularly in developing and among rural populations where men, women, and children all work and live near areas densely polluted with little or no regard for necessary environmental rules. Lead and cadmium are amongst the worst culprits in terms of environmental pollutants especially in developing countries. It has been reported that workers are routinely exposed to the toxic metal and are thus said to constitute about 0.9% of the total global health burden with majority of cases in developing countries [1, 2], between 0.5 and 1.5 million being due to occupational exposure [2]. Lead and cadmium poisoning are major potential public health problems throughout the world particularly in developing countries. Long-term exposure to these pollutants can increase the risk of developmental and reproductive disorders, immune system disorders, endocrine disruption, impaired nervous system function, cardiac diseases, and development of certain cancers while children are at higher risk from exposure than adults [3].

Occupational exposure to lead and cadmium is of utmost importance because of many vocations directly/indirectly associated with the metals. Hence, contact occurs through breathing in air that contains their particles and sometimes by direct ingestion. Environmental emissions containing lead and cadmium as in automobiles and recently uncontrolled use of generating plants in homes and organizations are also veritable sources of contact [4].

Several mechanisms have been established in the pathophysiology of lead and cadmium in humans. One of these is the generation of oxidants and reactive oxygen species (ROS). Reactive oxygen species propagate inflammatory processes from one organ system to another through the activation and release of various cytokines. These have been shown to result in tissue oxidative stress and multiple organ failure as in cardiotoxicity, neurotoxicity, hepatotoxicity, and nephrotoxicity [5]. Oxidative stress develops due to imbalance between free radical generation and the antioxidant defense system [6].

Lead and cadmium toxicities have been implicated in the pathogenesis of various disease conditions [7], and toxicity from exposure to leaded gasoline plays a salient role in the induction of free radicals which inadvertently leads to oxidative stress especially in subjects that are occupationally exposed. These risks are particularly prevalent in developing countries where there is often little information on the safe handling or transportation of pollutants including chemicals in industry and agriculture [8]. Accumulating evidence indicates that workplace exposure to lead has become a source of concern [1, 6]. The work of Abdusalam et al. [6] who reported a high prevalence of elevated blood lead levels among organized and roadside automobile technicians led credence to the vulnerability of this group of artisans.

In Nigeria, there have been some reports on oxidative stress in persons occupationally exposed to pollutants including mixed chemicals [9, 10]; however, few have narrowed down specifically on auto technicians who are occupationally exposed to lead and cadmium. This study was designed to evaluate the antioxidant status in various auto technicians exposed to lead and cadmium in Ibadan, Oyo State.

## 2. Subjects and Methods

A total number of sixty-five (65) apparently healthy male individuals (aged 18 to 65years) were selected for this project using a structured questionnaire. They were chosen based on specific inclusion criteria which included nonsmoking, no known chronic illness (e.g., hypertension and diabetes mellitus), and nonalcoholics.

Out of the above number, thirty-four (34) were occupationally exposed participants (cases) who have been working as auto technicians for at least two years either as workers or as apprentices. They were in four categories:

- Group 1: auto mechanics (16 men)
- Group 2: battery repairers (4 men)
- Group 3: panel beaters (8 men)
- Group 4: auto painters (6 men)

The remaining thirty-one (31) were members of staff/students of the University College Hospital, Ibadan, Oyo State, who were not auto technicians (male subjects); they served as controls.

Ethical clearance was obtained from the University of Ibadan/University College Hospital Joint Research Ethics Committee. Informed consent was duly obtained from each subject before a blood sample was taken.

## 3. Method

### 3.1. Specimen Collection

Ten milliliters of venous blood was collected from each subject into a sample bottle containing lithium heparin. The plasma obtained after centrifugation was stored at -20°C until analysis.

Random urine samples were collected from participants into plain dry universal bottles.

### 3.2. Analysis

Determination of total plasma peroxide was done in blood samples from cases and control participants using the FOX-2 method [11] while total antioxidant activity (TAC) was carried out using the ferric-reducing antioxidant power (FRAP) method as described by Kambayashi et al. [12]. Levels of cadmium and lead were determined in the blood and urine samples from the cases and controls using atomic absorption spectrophotometry (AAS).

A comparative analysis of TAC and the toxic metals was carried out using ratios of fractions of the parameters to establish the relativity of the analytes in the various groups of auto technicians. Statistical analysis was done using SPSS version 21.0. All data were expressed as mean ± SD with differences between groups compared using Students' *t*-test and ANOVA. Correlation analysis was done using Pearson's correlation coefficient.

Differences between cases and controls were accepted as significant at the *P* < 0.05 level.

## 4. Results

A comparative analysis of anthropometric parameters of the study participants showed no significant difference in mean weight, height, BMI, and systolic and diastolic pressures between auto technicians and the controls (*P* < 0.05) (Table 1).

However, a comparative analysis of biochemical parameters of the study participants showed significant differences in mean blood and urine lead (Pb) levels, mean urine cadmium (Cd) levels, total plasma peroxide (TPP), and oxidative stress index (OSI) (*P* ≤ 0.006, 0.018, 0.026, 0.001, and 0.001) in auto technicians and controls. Mean total antioxidant capacity (TAC) was not significantly different in the two groups (*P* ≤ 0.056) (Table 1).

**Figure 1:**
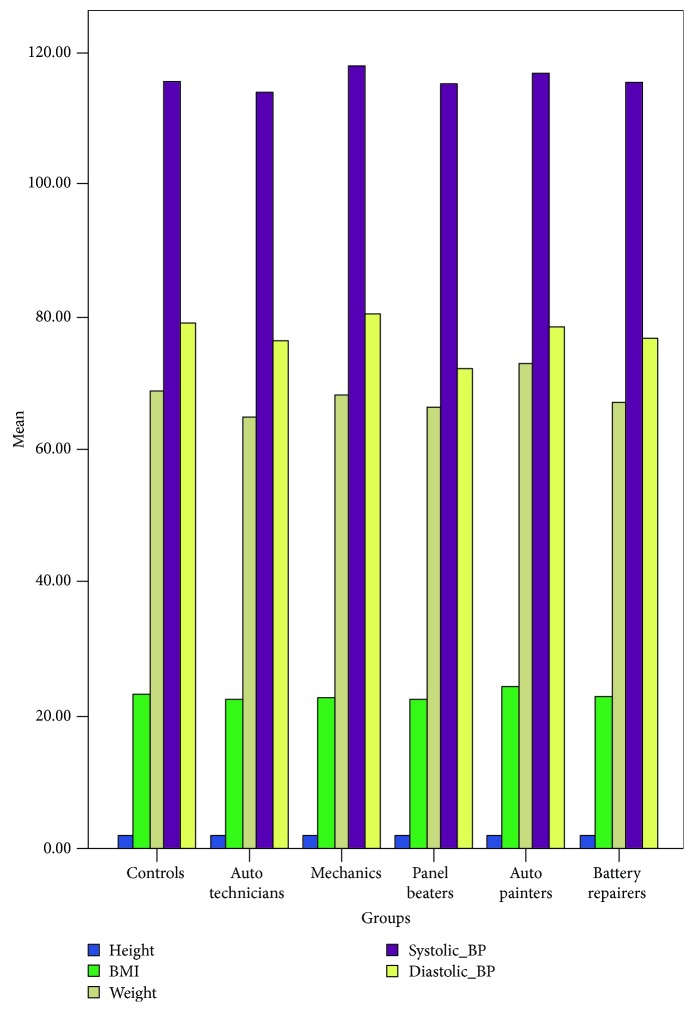
Bar chart showing anthropometric parameters between auto technicians and controls.

Towards stratification of the results amongst the subgroups of auto technicians studied, a comparative analysis of anthropometric parameters in the 4 subgroups of auto technicians (auto mechanic, panel beater, auto painter, and battery repairer) was done. There were no significant differences in these parameters in all the subgroups of auto technicians that participated in this study (*P* > 0.05) (Figure 1). This may justify the comparative analysis and deductions made earlier.

**Figure 2:**
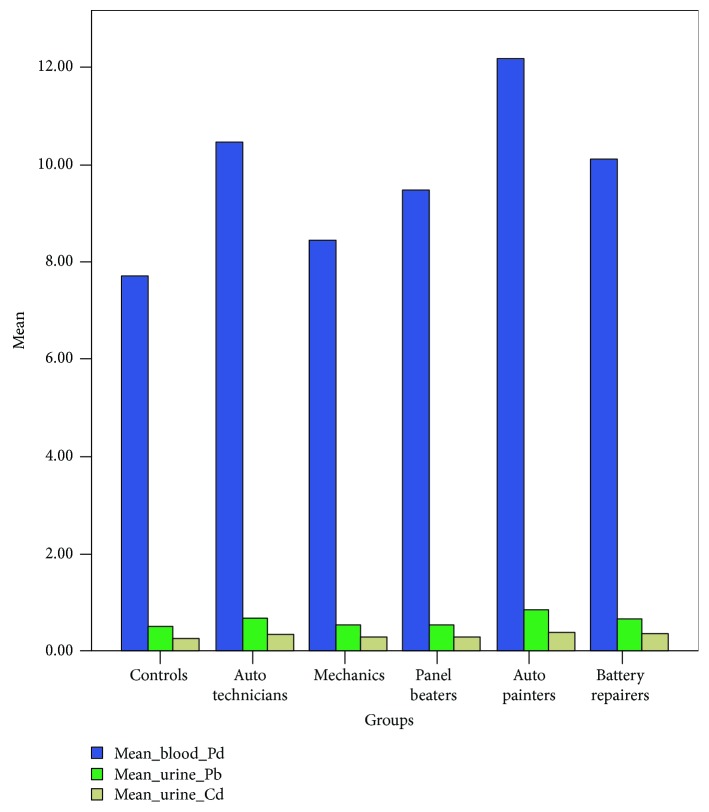
Bar chart showing comparative analysis of mean blood and urinary Pb and Cd levels in the subgroups of auto technicians relative to the control and the combined group.

Although there were no significant differences in the biochemical parameters of the various subgroups of auto technicians studied as shown by the mean blood Pb levels, it was apparent that battery repairers were most exposed to Pb compared to the others in the group. However, the clearance rate of Pb and Cd as shown by the mean urinary levels of the two toxic metals was also highest in battery repairers compared to other subgroups. Since urinary lead and cadmium levels are indicators of exposure to these metals in the presence of a functional renal system, the significantly increased urinary levels of Pb and Cd may be a serious indication of exposure to these toxic metals. Battery repairers were found to exhibit the highest level of excretion of the toxic metals, a phenomenon consistent with other results indicating that battery chargers are most vulnerable to oxidative distress occasioned by toxic metal exposure (Figure 2). Further comparative analysis showed that mean TAC was lowest in the same subgroup of battery repairers compared to other subgroups. Although in consonance with the above, the mean OSI index was also highest in battery repairers; however, it was observed that mean TPP concentration was highest in the descending order of panel beaters>auto mechanics>battery repairers>auto painters. This may indicate that the level of TAC notwithstanding, while battery repairers are more vulnerable to other diseases of lead and cadmium toxicities, panel beaters may be specifically more vulnerable to developing lipid peroxidation and its attendant diseases among the subgroups of auto technicians. Aside from this being consistent with the level of TAC, it may also indicate an increased vulnerability to oxidative damages generally as different from the specific TPP prognostic picture of lipid peroxidation as the pathophysiological pathway of diseases in this subgroup of auto technicians.

Correlation analysis of the biochemical parameters between controls and cases broadly showed that OSI expectedly correlated negatively but significantly with TAC in controls and cases and significantly positively with TPP in cases and controls. The significant positive correlation also observed between urinary Pb and urinary Cd in cases and controls may be yet another pointer to the increased exposure of auto technicians generally to the toxic metals in comparison with the nonoccupationally exposed controls. However, while a negative nonsignificant correlation was observed between TAC and blood Pb levels and between TPP and urinary Cd and Pb in cases, positive nonsignificant correlation was observed among these same parameters in controls. This discordant relationship may be a pointer to the possible pathophysiology of these toxic metals in cases different from in controls (Tables 2 and 3).

An analysis of ratios of these biochemical parameters against OSI in the various subgroups of auto technicians showed a marked reduction in the TAC : OSI ratio in all the subgroups in comparison to the controls. The distribution of values of these ratios were in this ascending order: battery repairer<auto mechanic<auto painter<panel beater. This clearly demonstrated the earlier observed relative vulnerability of the various subgroups of auto technicians to diseases associated with exposure to Pb and Cd, an inadvertent phenomenon in these occupations in this environment (Table 4).

Analysis of educational, domestic, dietary, and other ancillary exposures of participants showed that factors such as educational background, workplace environment, frequency of attendance at workplaces, and consumption of herbal preparations without any scientific basis of nutritional value were all significantly different between controls and cases. These factors may also exacerbate the development of various medical problems occupationally associated with these vocations (Table 5).

## 5. Discussion

Auto technicians belong to a group of subprofessionals whose training center on the repair and maintenance of the mechanical, electrical, and physical conditions of automobiles. Due to the necessity of automobile applications, their vocation is among those required for the daily economic growth of the society, hence their relevance. This group of artisans consists of several subgroups including auto mechanics, auto painters, auto electricians, battery chargers/repairers, panel beaters, and some others who all uniquely are trained to carry out specific functions in the maintenance and repair of auto vehicles. In the course of their vocation, they are generally exposed to several chemicals and pollutants some of which are known toxicants. Many reports have been written on their antioxidant profile [4, 13, 14]; however, there is little or no information (known to us) on the stratification of exposure of these subgroups of auto technicians and their differential vulnerability to an imbalance in oxidant/antioxidant levels. The need for this becomes imperative to enable a focused approach to management of diseases afflicting this group of artisans especially those relating to oxidant/antioxidant imbalance considering their relevance in the daily economic activity of any society. Generally, chemicals and metal toxicants like hydrocarbons, polyphenols, lead, and cadmium are among those to which auto technicians are occupationally exposed [15, 16]. It has been documented that this exposure can trigger several biochemical responses [17, 18]; among them is the oxidant/antioxidant pathway that may generate an imbalance depending on the tilt of the equation [19, 20]. This work was thus designed to stratify the relative effect(s) of exposure to these toxicants in the various subgroups of auto technicians.

In this study, average exposure to lead (Pb) was generally higher in all auto technicians compared to controls. Estimation of blood and urinary Pb and Cd excretion levels in cases and controls was meant to assess the extent of exposure to these toxic metals. Hence, the observed increased mean urinary excretion of Pb and Cd which correlated significantly in both auto technicians and controls may be indicative of excessive exposure to the toxic metals. That the metals were readily excreted may also eliminate the possibility of abnormality in the renal clearance system as a nonconfounding issue in the observed results. Although blood Cd level was not estimated in this study, however from the blood Pb level estimated, battery repairers were more exposed and therefore may be more vulnerable to the deleterious effects of Pb as a toxic metal than the other subgroups. In terms of Pb exposure, battery repairers were closely followed by auto mechanics and auto painters and the least exposed were the panel beaters in that descending order. That battery repairers constituted the most vulnerable in terms of exposure to lead may be informed by the uniqueness of their vocation among the other auto technicians since auto batteries contain a large amount of lead. Essentially, batteries for automobiles consist of a casing usually made of alloys of polycarbons, several cells or plates, and terminals both of which are essentially made of lead. These are suspended in an acid solution and sealed. The complete system generates the necessary electrical current that drives auto vehicles which gradually erodes the lead content of the cells and terminals and constantly emit fumes containing lead. The battery repairer is thus constantly exposed to the fumes in the course of his routine work which may have resulted in the observed elevated blood Pb levels in battery repairers in this study. In a similar work in Pakistan [21, 22], an increased exposure to lead by battery repairers among other auto technicians was also reported.

The auto mechanic's exposure to lead may be largely due to unhealthy and unethical habits in the course of their practice. This is because, essentially, contact with lead by this subgroup of auto technicians may largely be from leaded gasoline especially in this part of the world where leaded gasoline is still on sale [23, 24]. The unhealthy habit of sucking petrol with the bare mouth and using petrol to wash hands while working without further washing-out with soap and water before eating with these bare hands may also contribute to the source of exposure to the toxicant. Secondarily, contact may also be involuntarily from exhaust of cars and other automobiles especially in some of the mechanic garages where ventilation is poor. Hence, accumulation of lead in the workplace environment may be the most veritable source of contact with the toxicant through inhalation. Exposure of auto painters and panel beaters to excessive lead may be traceable to the content of vehicle paints, other chemicals used in the process (for auto painters), and lead content of the metal alloy or fabric used in the body of the vehicle (panel beaters). The possibility of indirect ingestion of excess amount of lead and other toxicants through inhalation by the two subgroups of auto technicians is largely informed by the lack of proper health education. This is because majority of these artisans find it very difficult to wear simple protective cloths and masks that may have protected them from lead-containing dust in their daily operations. Hence, their vulnerability is largely informed by this unethical habit and may be traceable to their level of education which may prevent their appreciation and compliance with benefits of various industrial rules.

Although blood cadmium level was not determined, evaluation of urinary cadmium and urinary lead may be an indication of degree of exposure to these metals. However, it may also reflect the clearance rate of the metals and by inference the efficiency of the renal system of participants. That urinary cadmium levels were similar in cases and controls may have indicated the effectiveness and therefore possibly the efficiency of kidney excretory function in the participants. It must however be pointed out that kidney dysfunction due to lead and/or cadmium exposure is an insidious process occurring at the renal tubular level, and because of the reserve capacity of the kidney, it may take a long time before it manifests [25, 26]. Hence, possibility of the covert presence of kidney tubular dysfunction may not be totally ruled out. There have been several reports on occupational exposure to lead such as welding, panel beating, lead ore smelting, and production of lead-containing pigments [6, 27]. These exposures have been reported to result in chronic poisoning which mostly affects enzymes and hematopoietic, peripheral, and nervous systems most of which may not manifest until the chronic stage when renal transplant will be inevitable [6].

Lack of or compliance with appropriate public and industrial health education in terms of wearing protective apparel while working, observing standard work ethics like not eating or smoking at work, and good hygienic practice of washing hands (and body when necessary) before eating may have also contributed to the observed elevated blood lead level in these artisans. Information on the educational attainment of most of these auto technicians derived from the questionnaire may be proof of their little or no education, hence the lack of appropriate public health knowledge that may enhance compliance with their work ethics.

The known effect of lead/cadmium toxicity on the oxidant/antioxidant balance of the body informed determination of the oxidant/antioxidant status of participants in this study. The observed nonsignificantly reduced total antioxidant capacity (TAC) of auto technicians compared to controls was more markedly shown by the highly significant elevation in total plasma peroxide (TPP) levels and increased oxidative stress index (OSI). However, stratification of the data among the various subgroups of auto technicians also indicated that the battery repairers may be the most vulnerable to problems of oxidative stress. The observed reduction in TAC may not be unrelated to their elevated blood Pb level while the stratification showing that battery chargers were the most incapacitated may also be further proof of the degree of vulnerability of this and each of the other subgroups to the degradation in oxidant/antioxidant balance in the course of their vocation. Correspondingly, OSI followed the same pattern as the TAC in the subgroup of auto technicians with the battery repairers having the highest OSI and following the same descending order as previously stated for TAC. Similar work [21, 22] showed that battery chargers were the most vulnerable to reduced TAC and increased OSI among the various subgroups of auto technicians studied. However, it is interesting to note that TPP was highest in panel beaters, the group with the lowest OSI among the subgroups. TPP is indicative of vulnerability to lipid peroxidation, an aspect of OSI, which is closely associated with membrane damage-facilitating cell membrane disruption which may possibly lead to mutagenicity or carcinogenicity in the subjects. It may therefore be inferred that while panel beaters are the least vulnerable to a reduced TAC generally among these auto technicians, they are the most vulnerable to lipid peroxidation, the end product of which may be mutagenesis or carcinogenesis.

An assessment of the malondialdehyde level in the subgroups of auto technicians would have been able to corroborate the TPP pattern since this oxidant (malondialdehyde) is a good indicator of possible mutagenicity or carcinogenicity due to proliferating lipid peroxidation [28]. Unfortunately, estimation of level of this oxidant was not included in this study.

Evidences abound that there are myriads of interactions between heavy metals and living tissues which could invariably alter the oxidant/antioxidant balance of the body [29]; many pathological conditions have been attributed to this mechanism including toxic metal-induced cancer. The associated symptoms owing to the gradual accumulation of toxic metals are multiple and rather nondescript. Overt expression of deleterious effects may not appear until later in life [15, 16]. It may therefore be inferred that these pathological changes are inherent in daily vocations exposed to Pb and Cd.

## 6. Conclusion

Oxidative stress exists in individuals who work as auto technicians (panel beaters, auto mechanics, battery repairers, and auto painters) as they are constantly occupationally exposed to heavy metals which include lead and cadmium. From this study, increased concentration of lead and cadmium was observed in auto technicians recruited in Ibadan, Oyo State. Exposure to lead can induce anemia both by reducing the survival of red cells through increased membrane lipid peroxidation or interfering with heme biosynthesis. Other mechanisms by which both lead and cadmium induce toxicity are by causing antioxidant/oxidant imbalance which may also enhance development of chronic diseases like cancer.

## 7. Recommendation

Adequate sensitization and public/industrial health education towards ensuring understanding of the potential hazard of the various chemicals and metal pollutants they are exposed to is salient in the prevention and amelioration of the insidious lead- and cadmium-induced oxidative stress. Thorough hand washing before eating and wearing of protective apparel at work particularly nose masks to reduce the inhalation of toxic fumes are vital for auto technicians. Regular biomonitoring is important in the prompt and early amelioration of exposure to lead and/or cadmium to abate its toxicity. These preventive measures may help in reducing the risk of developing diseases associated with exposure to toxic levels of Pb and Cd and their attendant chronic diseases associated with degradation in oxidant/antioxidant balance.

## Figures and Tables

**Table 1:** Anthropometric and biochemical parameters of auto technicians and controls.

Parameters	Controls *N* = 31	Auto technicians *N* = 34	*t*-test	*P* value
Weight	68.65 ± 12.63	67.08 ± 9.16	0.578	0.567
Height	1.73 ± 0.07	1.72 ± 0.70	0.506	0.614
BMI	22.91 ± 3.45	22.70 ± 2.95	0.269	0.789
Systolic BP	115.26 ± 4.61	115.44 ± 8.80	-0.104	0.918
Diastolic BP	79.03 ± 5.77	76.91 ± 7.64	1.252	0.215
Blood Pb	7.72 ± 1.22	10.11 ± 4.47	-2.871	**0.006** ^∗^
Urine Pb	0.52 ± 0.19	0.65 ± 0.21	-2.422	**0.018** ^∗^
Urine Cd	0.27 ± 0.10	0.34 ± 0.11	-2.275	**0.026** ^∗^
TAC	26741.87 ± 8696.68	22538 ± 8726.54	1.943	**0.056**
TPP	120.16 ± 70.54	183.88 ± 53.39	-4.128	**0.001** ^∗^
OSI	0.49 ± 0.33	0.93 ± 0.45	-4.373	**0.001** ^∗^

^∗^Significant at *P* ≤ 0.05.

**Table 2:** Correlation between biochemical parameters in control participants.

	Blood lead	Urine lead	Urine cadmium	TAC	TPP	QSI
	*r* (*P*)	*r* (*P*)	*r* (*P*)	*r* (*P*)	*r* (*P*)	*r* (*P*)
Blood lead	1					
Urine lead	0.021 (0.909)	1				
Urine cadmium	-0.087 (0.642)	0.924 (0.001)^∗^	1			
TAC	0.014 (0.940)	0.103 (0.580)	0.153 (0.410)	1		
TPP	0.118 (0.527)	0.172 (0.356)	0.147 (0.430)	-0.018 (0.924)	1	
OSI	0.204 (0.270)	0.120 (0.520)	0.113 (0.546)	-0.399 (0.026)^∗^	0.860 (0.001)^∗^	1

^∗^Significant at *P* ≤ 0.05. This table shows that urine lead and urine cadmium are significantly positively correlated (*r* = 0.924, *P* ≤ 0.001) in control participants. Also, there is a positive correlation between OSI and TPP (*r* = 0.860, *P* ≤ 0.001) while OSI and TAC are negatively correlated (*r* = ‐0.399, *P* = 0.026).

**Table 3:** Correlation between biochemical parameters in automobile technicians.

	Blood lead	Urine lead	Urine cadmium	TAC	TPP	OSI
	*r* (*P*)	*r* (*P*)	*r* (*P*)	*r* (*P*)	*r* (*P*)	*r* (*P*)
Blood lead	1					
Urine lead	0.609 (0.699)	1				
Urine cadmium	-0.043 (0.808)	0.975 (0.001)^∗^	1			
TAC	-0.119 (0.501)	0.024 (0.891)	0.065 (0.714)	1		
TPP	0.001 (0.996)	-0.016 (0.927)	-0.078 (0.661)	0.072 (0.687)	1	
OSI	0.040 (0.821)	0.018 (0.920)	0.043 (0.810)	-0.591 (0.001)^∗^	0.587 (0.001)^∗^	1

^∗^Significant at *P* ≤ 0.05. This table shows that there is strong positive correlation between urine lead and urine cadmium in auto technicians (*r* = 0.975, *P* ≤ 0.001). OSI and TAC are negatively correlated (*r* = ‐0.591, *P* ≤ 0.001) while OSI correlates positively with TPP (*r* = 0.587, *P* ≤ 0.001).

**Table 4:** Comparative analysis of ratios of OSI with Pb, Cd, TAC, and TPP in the various auto technicians and controls.

	Control	Cases	1	2	3	4
Urinary Pb : OSI	1.06 ± 0.55	0.69 ± 0.47	0.7340 ± 0.53	0.64 ± 0.62	0.62 ± 0.29	0.75 ± 0.25
Urinary Cd : OSI	0.55 ± 0.30	0.37 ± .24	0.38 ± 0.26	0.33 ± 0.32	0.35 ± 0.19	0.36 ± 0.19
TAC : OSI	54573.47 ± 26354.55	24234.41 ± 19393.33	23579.79 ± 20142.54	29815.48 ± 21110.81	25743.82 ± 10577.94	16627.03 ± 10913.89
TPP : OSI	245.22 ± 213.75	197.85 ± 117.76	197.87 ± 163.65	245.24 ± 105.41	186.52 ± 104.41	149.55 ± 50.00

Legend: 1: auto mechanic; 2: panel beater; 3: auto painter; 4: battery repairer.

**Table 5:** Characteristics of participants in the study.

Variables	Controls	Frequency (%)	Auto technicians		*P*
	*N*		*n*	Frequency (%)	
*Education*					**0.001**
Graduate	17	54.8	0	0	
Post graduate	7	22.6	0	0	
Senior school cert	2	6.5	17	50.0	
Undergraduate	5	16.1	0	0	
Primary school	0	0.0	12	35.8	
Junior secondary	0	0.0	4	11.8	
No formal education	0	0.0	1	2.9	
*Ethnic group*					0.051
Yoruba	26	83.9	34	100	
Igbo	3	9.7	0	0	
Hausa	0	0	0	0	
Others	2	6.5	0	0	
*Work environment*					**0.001**
Indoor	31	100	20	0	
Workshop	0	0	14	58.8	
Outdoor	0	0	0	42.2	
*Alcohol consumption*					0.304
Yes	8	25.8	13	38.2	
No	23	74.2	21	61.8	
*Herbal concoction consumption*					**0.001**
Yes	16	51.6	32	94.1	
No	15	48.4	2	5.9	
*Analgesic use*					0.427
Yes	15	55.6	23	67.6	
No	12	44.4	11	32.4	
*Multivitamin use*					1.000
Yes	18	62.1	21	63.6	
No	11	37.9	13	36.4	
*Days of work*					**0.001**
5 days	23	74.2	4	11.8	
6 days	6	19.4	27	79.4	
7 days	2	6.5	3	8.8	
*Drug use*					4.93
Yes	0	0	2	5.9	
No	31	100	32	94.1	
*Fruit and vegetable*					0.065
Occasionally	8	25.8	14	41.2	
Daily	18	58.1	10	29.4	
Weekly	5	16.1	10	29.4	

## Data Availability

All data in support of this work are included in the manuscript. They can also be obtained by contacting the corresponding author at the email provided on the manuscript. The data are freely available.
